# Primary care provision for young people with ADHD: a multi-perspective qualitative study

**DOI:** 10.3399/BJGP.2023.0626

**Published:** 2024-04-30

**Authors:** Rebecca Gudka, Kieran Becker, John Ward, Jane R Smith, Faraz Mughal, GJ Melendez-Torres, Tamsin Newlove-Delgado, Anna Price

**Affiliations:** University of Exeter Medical School, University of Exeter, Exeter.; University of Exeter Medical School, University of Exeter, Exeter.; University of Exeter Medical School, University of Exeter, Exeter; Department of Psychiatry, University of Oxford, Oxford.; University of Exeter Medical School, University of Exeter, Exeter.; School of Medicine, Keele University, Keele.; University of Exeter Medical School, University of Exeter, Exeter.; University of Exeter Medical School, University of Exeter, Exeter.; University of Exeter Medical School, University of Exeter, Exeter.

**Keywords:** ADHD, prescribing, primary care, qualitative research, shared care

## Abstract

**Background:**

Attention deficit hyperactivity disorder (ADHD) is a highly prevalent neurodevelopmental disorder. UK guidance states that primary care has a vital role in effective ADHD management, including referral, medication prescribing and monitoring, and providing broader mental health and wellbeing support. However, many GPs feel unsupported to provide health care for young people with ADHD. Inadequate health care is associated with rising costs for patients and society.

**Aim:**

To investigate the experiences of young people with ADHD accessing primary care in England, from the perspectives of people with lived experience of ADHD and healthcare professionals (HCPs).

**Design and setting:**

A qualitative study. Interviews were conducted with HCPs (GPs, practice managers, and a wellbeing worker) and people with lived experience of ADHD (young people aged 16–25 years and their supporters) located in integrated care systems across England.

**Method:**

Semi-structured interviews were conducted with participants at five purposively selected general practices (varying by deprivation, ethnicity, and setting). Questions focused on experiences of accessing/providing health care for ADHD. Reflexive thematic analysis was undertaken within a critical realist framework to understand how provision works in practice and to explore potential improvements.

**Results:**

In total, 20 interviews were completed with 11 HCPs and nine people with lived experience. Three themes were generated: a system under stress, incompatibility between ADHD and the healthcare system, and strategies for change in ADHD primary care provision.

**Conclusion:**

Standardisation of ADHD management in primary care, providing better information and support for HCPs, and advising on reasonable adjustments for people with lived experience could help improve access to effective treatments for young people living with ADHD.

## Introduction

Attention deficit hyperactivity disorder (ADHD) affects an estimated 3%–5% of children and adolescents, and 2%–5% of adults globally.^1^^–^4 Nearly half of individuals diagnosed with ADHD during childhood continue to experience symptoms into adulthood.^5^ ADHD can predispose young people to develop mental health problems, and lead to negative long-term outcomes such as worse academic and employment opportunities, financial difficulties, higher engagement in criminal activity, and increased mortality.^6^^–^8 Treatment options include medication and non-pharmacological support, such as psychosocial interventions. Both offer short-term efficacy in ameliorating ADHD symptoms,^9^^,^^10^ while medication has been shown to improve long-term outcomes.^6^ Providing patients with adequate access to support, including medication where needed, enables effective management of ADHD.^11^^,^^12^

GPs and primary care are the interface between patients and specialist services in the UK. Under National Institute for Health and Care Excellence (NICE) ADHD guidelines,^11^ primary care professionals can refer patients for assessment/diagnosis and prescribe medication under shared care agreements. However, research indicates patchy provision, with long waiting times and limited availability of adult ADHD services.^13^^,^^14^ Often, the components needed to enable shared care prescribing of ADHD medication are not in place.^15^ Additionally, research indicates that shared care does not work consistently in practice, with concerns over the balance of risk and responsibility.^15^^,^^16^

Previous qualitative research has examined stakeholder experiences of ADHD management in primary care from the perspectives of parents and older adults.^17^ However, young people face additional challenges as they transition into adulthood.^18^ Appropriate support in this period is critical to maintain engagement with treatment and for future mental health. Therefore, this study aimed to explore the experiences of young people (aged 16–25 years) with ADHD when accessing primary health care, incorporating perspectives of young people with lived experience of ADHD and their supporters, and primary healthcare professionals (HCPs). The objectives were to understand how young people with ADHD access appointments and receive care for ADHD, including referrals, prescriptions, and reviews/monitoring; to identify the types of support available from primary care for young people with ADHD; and to explore barriers to accessing support for ADHD in primary care.

**Table:** How this fits in

Attention deficit hyperactivity disorder (ADHD) is a highly prevalent neurodevelopmental disorder, with negative consequences for individuals and their communities. Research indicates a current failure of health care for people with ADHD in England, but previous recommendations to improve support for ADHD in primary care lack feasible and practical recommendations for healthcare professionals. This study highlights individual-, practice-, and system-level barriers to accessing support for ADHD via primary care, and provides suggestions for how to overcome these barriers from the perspectives of multiple stakeholders. Healthcare professionals and people with lived experience of ADHD provided data, which points to the standardisation of ADHD provision, providing additional information and support for clinicians, and better use of reasonable adjustments for patients with ADHD in general practice.

## Method

### Participants and recruitment

Previously, the Managing young people (aged 16–25) with ADHD in Primary care study^19^ investigated the provision of supportive elements for ADHD in primary care, reported by people with lived experience and HCPs. Potential primary care practice sites were identified via MAP survey responders and purposively sampled from across England to represent a range of local area characteristics (ethnicity, setting, and deprivation). Participants were recruited via practice sites or participation in the national survey where they indicated a location close to one of the sites. All participants had to be currently residing or working in England. Further detail of our recruitment strategy can be found in the MAP study protocol.^19^

### Data collection

Data were collected using semi-structured interviews with topic guides (see Supplementary Information S1 for details). Topic guides were initially developed using questions from the MAP survey, and refined following consultation with two research advisory groups, whose members were recruited to support the wider MAP study.^19^ A young person research advisory group included young people with ADHD and their supporters, and a practice and policy research advisory group consisted of practitioners and service commissioners. Questions focused on experiences of accessing/providing health care for ADHD. Box 1 gives summaries of the topic guides.

**Box 1.** Summary of topic guides

**Health professionals and providers**
**1. Access to primary care for ADHD**
Experiences of how young people with ADHD access primary care, including practicalities, barriers, and facilitators
**2. Support for primary care providers working with young people and adults with ADHD**
Thoughts on how local providers and wider healthcare system can support primary care professionals to provide health care for ADHD, including challenges and changes that can be made to the system
**3. Providing care for people with ADHD through primary care**
Experiences of providing care in the following domains:
Prescribing and medication — shared care protocols and differences in private/NHS diagnosis for prescribing
Non-pharmacological ADHD health care — mental health support, social prescribing, and transition to adult mental health services
**4. Wider mental and physical health support through primary care**
Experiences providing for the wider health needs of young people and adults with ADHD, including health risks associated with ADHD and the Additional Roles Reimbursement Scheme
**5. Information resources and digital solutions**
Knowledge and use of digital health interventions for young people with ADHD, and digital resources that aid practice and delivery of primary care

**Young people and supporters**
**1. Access to primary care for ADHD**
Experiences of how young people with ADHD access primary care, including practicalities, barriers, and facilitators
**2. Support for young people and adults with ADHD through primary care**
Experiences of receiving care in the following domains:
Referral for ADHD diagnosis — via NHS or private routes (including Right to Choose)
Prescribing and medication — how prescriptions are set up/received, communicating between adult mental health services and GPs, and medication reviews
Non-pharmacological ADHD health care — mental health support and transition to adult mental health services
**3. Wider mental and physical health support through primary care**
Experiences receiving care for wider health needs/comorbidities, including awareness of health risks associated with ADHD
**4. Information resources and digital solutions**
Knowledge and use of digital health interventions for self-management of ADHD symptoms or information about accessing health care. Preferences with regards to future delivery of digital health intervention

*ADHD = attention deficit hyperactivity disorder.*

Interviews took place between March and June 2023 either online via Microsoft Teams or via telephone, with informed consent. All interviews were audio-recorded and transcribed verbatim by an approved third party. All identifiable participant information was anonymised. Recordings and transcripts were stored on a general data protection regulation-compliant server only accessible by members of the research team.

### Data analysis

A reflexive thematic analysis was conducted to generate themes, as described by Braun and Clarke,^20^ and exemplified by Byrne.^21^ This was undertaken within a critical realist framework,^22^ using NVivo (version 14) to manage the data. A preliminary inductive framework was created by immersion in the interviews and line-by-line coding of two transcripts by two researchers. This was then revised, incorporating deductive codes from the topic guides. Remaining transcripts were coded by three researchers, supported by regular meetings to discuss and refine the framework with the wider team. Coders maintained reflective journals throughout, documenting their personal perspectives. Column summaries were created for the framework matrix, then organised into themes and subthemes, which were applied to the data and refined by four researchers.

## Results

### Sample

In total, 20 participants (11 HCPs and nine people with lived experience) were recruited from five primary care practices in England, located across five of the seven NHS regions. Practice location characteristics are outlined in Table 1 and individual participant details are given in Table 2.

**Table 1.** Overview of research sites, participants, local area profiles, and site descriptions

**Practice research site (number of participants)**	**Profile of local authority**			**Profile of practice, including characteristics of local area as described by participants**	
	**% ‘White’^a^**	**Rural/urban classification^b^**	**IMD rank^c^**	**Summary**	**Quotes**
S1 (*n* = 3)	94.0	Urban with city and town	50	∼12 500 patients. High deprivation area. Specialists in substance misuse. Contract for homeless outreach. Social prescriber and MH worker.	*‘Most deprived practice in the city … top 10% of most deprived practices in the country. We see a huge amount of mental health, substance misuse. We’re specialists in substance misuse … We hold the contract for the Outreach to the Homeless Service, and we take our care out to hostels*.*’* (PracticeManager-1) *‘We have a social prescriber through the Additional Role*[s] *Reimbursement Scheme, with the PCNs … that works in our practice … We’ve got a mental health worker*.*’* (PracticeManager-1)
S2 (*n* = 5)	84.0	Urban with major conurbation	1	∼3500 patients. High deprivation area. Provide drug misuse services and SAS services. Counsellor.	*‘One of the areas of England with the highest index of multiple deprivation.’* (PracticeManager-2) *‘Inequalities … massive … lack of job opportunities, education, housing, you name it … high asylum seeker community … a really, really, diverse area.’* (PracticeManager-2) *‘We provide a drug misuse service … SAS service … for patients who have been removed from their practice having behaved in a way that warranted the police being called*.*’* (GP-2) *‘We have an onsite counsellor who’s available for brief interventions*.*’* (GP-2)
S3 (*n* = 4)	97.6	Largely rural	75	∼7000 patients. Link workers and MH workers.	*‘We have link workers, and … primary mental health workers*.*’* (GP-3)
S4 (*n* = 4)	70.7	Urban with city and town	180	∼7000 patients. University-linked practice. High numbers of students and patients from overseas. Wellbeing worker.	*‘We have a young population … we also have a lot of overseas families and students.’* (PracticeManager-4) *‘We have a …* [wellbeing] *worker who comes to the practice who doesn’t provide therapy as such but is very good for exploring complex issues and kind of way finding and thinking about other resources that may be needed or accessible to a particular patient*.*’* (GP-4)
S5 (*n* = 4)	48.6	Urban with major conurbation	7	∼5500 patients. High deprivation area. MH worker and social prescriber.	*‘High deprivation area … one of the most deprived communities in … in England, so lowest 10%.’* (GP-5) *‘We have a mental health worker … social prescribers*.*’* (PracticeManager-5)

a *2021 Census, percentage of people classing themselves as ‘White’, with the average in England being 81.0%.^23^*

b *2011 Census.^24^*

c *Indices of Multiple Deprivation (IMD) summaries for local authority districts;^25^ rank of proportion of lower layer super output areas in most deprived 10% nationally, with 1 being the highest rank for deprivation and 195 being the lowest. MH = mental health. PCN = primary care network. SAS = Special Allocation Scheme.*

**Table 2.** Participants: unique identifier, role, and additional characteristics

**Unique identifier**	**Site**	**Role**	**Sex**	**Ethnicity (self- described)**	**Additional information (provided at interview)**
PracticeManager-1	1	Practice manager	Female	White British	Managing partner
YoungPerson-1	1	Young person with ADHD^a^	Male	White British	Aged 20 years, student
YoungPerson-2	1	Young person with ADHD^a^	Female	White British	Aged 24 years, graduate
GP-1	2	GP^a^	Female	White British	Commissioning experience/role, ADHD in family
GP-2	2	GP	Male	White British	ADHD in family
PracticeManager-2	2	Practice manager	Female	White British	Possible undiagnosed ADHD, ADHD in family
YoungPerson-3	2	Young person with ADHD	Female	White	Aged 24 years, mother
Supporter-1	2	Supporter of young person with ADHD	Male	White British	Grandfather
YoungPerson-4	3	Young person with ADHD^a^	Male	White British	Aged 17 years, apprentice
Supporter-2	3	Supporter of young person with ADHD^a^	Female	White British	Mother
GP-3	3	GP	Female	British mixed	Commissioning experience/role, ADHD in family
PracticeManager-3	3	Practice manager	Female	White British	—
PracticeManager-4	4	Practice manager	Female	White	—
WellbeingWorker	4	Wellbeing worker	Female	White other	Neurodiverse
GP-4	4	GP	Male	Irish	—
YoungPerson-5	4	Young person with ADHD	Female	White British	Aged 24 years, student
Supporter-3	5	Supporter of young person with ADHD^a^	Female	White British	Mother
YoungPerson-6	5	Young person with ADHD^a^	Female	White British	Aged 22 years, on a break from university
PracticeManager-5	5	Practice manager	Female	British Pakistani	—
GP-5	5	GP	Male	British Pakistani	Commissioning experience/role

a *Participant recruited via Managing young people (aged 16–25) with ADHD in Primary care study^19^ (instead of via site) from location within same local area as practice research site. ADHD = attention deficit hyperactivity disorder.*

### Findings

Three themes were generated relating to primary care provision for young people with ADHD: a system under stress, incompatibility between ADHD and the healthcare system, and strategies for change. These are summarised in Box 2.

**Box 2.** Summary of themes, with illustrative quotes

**Theme and sub-themes**	**Summary**	**Illustrative quotes**
A system under stress: Lack of provision Deferring responsibility of care Variation in ADHD management	Demand for ADHD services is increasing, stretching already limited resources even further. A lack of capacity in primary care, coupled with gaps in secondary care, negatively impacts care pathways. Patients and healthcare professionals reported difficulties linked with variations in ADHD management, within and between practices.	*‘The health service in general practice particularly is not paid to support everything. We have limited resources; we have to manage within those limited resources*.*’* (GP-5) *‘The particular one is the complete absence, or the paucity of actual services for people with ADHD. So, it’s great when I can see somebody with angina. I know I’ve got a system where I can assess that patient, I can arrange investigations. I can pull together an initial primary care treatment programme and I can refer on … and I know that system’s going to work. I don’t have that for ADHD. The systems are very poor*.*’* (GP-2)
Incompatibility between ADHD and the healthcare system: Barriers to accessing care — a complex system Consequences for the individual	The systems/processes involved in accessing care are counterintuitive to characteristics of ADHD, such as difficulties with organisational skills and attention. These barriers have consequences for individuals.	*‘But I think just things like inflexible appointment keeping and being thrown off waiting lists because you are not keeping your appointments or making it very difficult for you to enter systems because you have got to fill in very big questionnaires and submit them on time, that kind of thing, that can be hard for people*.*’* (GP-1)
Strategies for change in ADHD primary care provision: Clarifying responsibility for care Need for training/information Improving provision	Three main areas of focus for change are identified to best equip healthcare professionals to provide care to young people with ADHD: clarifying responsibility, providing training and information, and improving provisions	Example of good practice: *‘I think what we provide is having quite a knowledgeable practice workforce, who see a lot of patients with ADHD. They have got understanding and knowledge. We provide longer appointments … that would be helpful.’* (PracticeManager-4)

*ADHD = attention deficit hyperactivity disorder.*

### A system under stress

#### Lack of provision

Many participants described difficulties accessing prescriptions for ADHD medication from their GP, especially if they had been diagnosed privately. This is of concern given that several participants with lived experience reported that they *‘ended up with a private diagnosis because of the* [NHS] *wait times’* (YoungPerson-2):

*‘So, my GP has refused to take over my prescriptions, so I still get them from Psychiatry UK. They didn’t give me a reason, they just said that they won’t do shared care.’*

(YoungPerson-1)

Additionally, participants with lived experience overwhelmingly reported receiving no medication monitoring from their GP, with one exception whereby the participant received check-ups, albeit at seemingly random intervals:

*‘I have basically no communication with them while I have the repeat prescription. Because it is all semi-automatic. And then, once in a blue moon, however much time has passed, doesn’t seem to have a pattern, they’ll not fill it, and go, “Oh, you actually need to come in, or you need to do your blood pressure and weight at our machine, and just send it to us.”’*

(YoungPerson-2)

Furthermore, HCP and participants with lived experience reported that *‘there is very little out there’* (PracticeManager-1) with regards to non-pharmacological support for ADHD or mental health from primary care. Many participants with lived experience stated that they *‘wouldn’t even know where to go at the GP’* (YoungPerson-4) to find such support:

*‘We get nothing through our doctor’s surgery. There are no support groups, there’s no specialised nurse there. It’s literally you get* [the] *prescription and that’s it.’*

(Supporter-2)

This was acknowledged by most HCPs, although there were some exceptions, with a university practice reporting strong mental health and welfare provision:

*‘Within the university setting there’s often a very strong welfare provision as well as a counselling service if needed, so students here are often very well supported from that point of view.’*

(GP-4)

Some practices offered access to mental health support but with *‘nothing specific to ADHD’* (GP-1), experiences echoed by both HCP and participants with lived experience*.* Many HCPs mentioned access to social prescribers who offer *‘generic support for people’* (GP-1). One GP provided examples of social prescriber assistance:

*‘Support with learning, support with any issues they might have with housing issues, help with benefits … food banks, that kind of stuff. But that’s not specific to ADHD.’*

(GP-3)

Some participants with lived experience felt that, while comorbidities or symptoms caused by their ADHD (such as low mood) might be recognised and treated, the underlying cause (ADHD) was not:

*‘Throughout the course of all of* [my] *treatments, I had always said it felt like we were treating a symptom, not the cause … It just feels like the foundation of what the problem is somehow is not being addressed*.*’*

(YoungPerson-6)

#### Deferring responsibility of care

Most participants with lived experience expressed feeling *‘pushed from pillar to post’* (Supporter-1) between different NHS services when accessing care. Many described trying Right to Choose (for example, via Psychiatry UK*,* a private psychiatry service that holds contracts with the NHS) for accessing referrals to mitigate long NHS waitlists, but HCPs pointed out that increased demand via these routes was deferring/moving waitlists:

*‘I’ll be looking to refer them to Psychiatry UK but that could well be a 9-month process; despite the best of intentions, their waiting lists are growing hugely, I believe*.*’*

(GP-2)

A concern expressed by some HCPs was that to mitigate for long waitlists they were being asked to *‘mop up’* (PracticeManager-3) after secondary care without recognition of additional responsibilities they were taking on:

*‘It becomes something else that once upon a time was managed by secondary care, and it suddenly becomes a primary care thing. That’s great in many ways, but it’s not great in primary care because we’re picking up something else from secondary … Maybe there needs to be recognition of that.’*

(PracticeManager-1)

#### Variation in ADHD management

HCPs discussed variation within and between practices in terms of shared care, prescribing practice, private versus NHS diagnoses, and knowledge or willingness of individual practitioners to prescribe:

*‘There is no universal way that people put shared care drugs on a prescribing system*.*’*

(GP-1)

*‘It is different, yes. So, with someone with an NHS diagnosis, there’s a fairly standard procedure; they titrate them, they get them stable, and then take over the actual prescribing … for people with a private diagnosis, that’s entirely different*.*’*

(GP-3)

One reported consequence was overwhelming demand at practices with *‘neurodiverse friendly’* (Supporter-2) systems, including multiple ways to book appointments or ability to email GPs:

*‘We do attract patients from other practices who are unable to get support and care, and there are some practices who will not accept shared care agreements from private providers.’*

(PracticeManager-4)

People with lived experience discussed negative impacts related to variations in care, including *‘no continuity’* (Supporter-3) within practices:

*‘But when you don’t see anyone who’s a regular in the practice … it’s sort of like “Okay, I’ll pass it on, pass it on”, and it sort of goes under the radar then.’* (Supporter-1)

### Incompatibility between ADHD and the healthcare system

#### Barriers to accessing care — a complex system

The most described barrier to accessing primary care was the complexity of the systems/processes involved. Many participants with lived experience found appointment booking to be a *‘frustrating’* (Supporter-3) process:

*‘You have to constantly keep prompting them. But if you’re getting through to your GP, you’ve got to ring from 8:00 and if you’re not through in time and there are no appointments left, you then have to ring back at 12:00 and see if there are any appointments left, and if there’s none left then you start again at 8:00 the next day, and it’s a circle like that*.*’*

(Supporter-2)

Both participants with lived experience and HCP found navigating the *‘whole rigmarole’* (GP-3) of referral processes, medication titration, and supporting transitions between child and adult services to be a highly complex process. Services were described as *‘work*[ing] *as two separate entities rather than together’* (Supporter-2):

*‘There’s a lengthy process from the patient attending their first appointment with the GP, like the process with regards to the referrals and communication, etc., with secondary care … I think sometimes it’s not clear who’s actually responsible for what*.*’*

(PracticeManager-3)

ADHD medication being a controlled substance created another reported barrier for patients, with the need for frequent repeat prescriptions. Shared care protocols were described as a *‘minefield’* (PracticeManager-3). Participants reported that this made changes to medication type and dose difficult:

*‘That has been a bit of an arduous process, in all honesty. Getting stuff moved onto repeat … having to continually follow up on that process*.*’*

(YoungPerson-6)

HCPs and people with lived experience identified that the healthcare systems people with ADHD must navigate were often incompatible with difficulties associated with ADHD, creating barriers for patients accessing care:

*‘It is more of a challenge for* [people with ADHD] *to keep to structures and appointments by systems that may not necessarily have much sympathy with their particular difficulties.’*

(GP-1)

*‘One of the main barriers … is the fact that the ADHD referral process … is not very ADHD-friendly, which seems sort of counterintuitive*.*’*

(YoungPerson-6)

#### Consequences for the individual

Several HCPs and participants with lived experience spoke about the mental health cost of living with ADHD and trying to access care, including reports of self-harm and suicidality:

*‘Just general self-neglect as a consequence of depression associated with ADHD … can lead to suicidal ideation and potential suicide risk or pseudo-suicide risk, and all of which can cause harm.’*

(GP-2)

Some participants with lived experience referred to an unofficial *threshold for care*, with exceptional levels of distress apparently needed to access secondary care, making positive management of their condition difficult:

*‘Why should it have to get to that point before you see someone? You need to get there before to prevent that.’*

(YoungPerson-4)

### Strategies for change in ADHD primary care provision

#### Clarifying responsibility for care

HCPs and participants with lived experience agreed that clarification or standardisation of responsibilities for provision would be helpful. Some felt that ADHD should be treated similarly to other chronic health conditions, with regular checks and clear guidelines/pathways, or an *‘ADHD nurse at the practice’* (Supporter-2):

*‘You have clear guidance and protocols for managing COPD* [chronic obstructive pulmonary disease] *and asthma, and all these other conditions, so maybe there needs to be one for something like that if it’s going to be managed in primary care, so we’re very clear what we have to do annually, 6-monthly, but it’s funded*.*’*

(PracticeManager-1)

However, there was disagreement about which services should manage ADHD:

*‘So maybe if* [ADHD] *became something managed in primary care, and people pop in and have their asthma review and their smear and diabetic review, they could pop in and have their ADHD review. That might normalise it a bit more, take off some of the stigma*.*’*

(PracticeManager-1)

*‘I really think that, asking us to prescribe medication for a condition technically they’re managing shouldn’t be at the request of the GP. I wonder why it’s not all just undertaken with secondary care.’*

(PracticeManager-3)

ADHD was not viewed as a disability or mental health condition by some HCPs; however, it did not fall into the category of physical health. This uncertain status as a condition resulted in some participants with lived experience feeling that ADHD was not seen as *‘important’* or *‘urgent’* (Supporter-2), and was a low priority for primary care:

*‘We wouldn’t necessarily routinely make reasonable adjustments around someone with ADHD like we would for someone with a learning disability or a severe mental illness or autism*.*’*

(GP-3)

#### Need for training/information

Many participants expressed a need for improved training and information, such as on care plans/pathways and medication, to help them feel more confident in caring for patients with ADHD:

*‘Neurodiversity and ADHD, it just needs to be covered as a base just for, like, every GP.’*

(YoungPerson-4)

Furthermore, both groups mentioned the importance of raising awareness of all ADHD traits, with some participants with lived experience mentioning HCPs missing inattentive symptoms, especially in female patients:

*‘But I do think one of the major barriers for me in terms of accessing support, was awareness of female/inattentive presentations of ADHD. That was a major … and it wasn’t until like, the 19th appointment or whatever it was*.*’*

(YoungPerson-5)

Key roles specifically mentioned by both stakeholders were those of reception staff. Participants reflected that a better understanding and tolerance of people with ADHD would be beneficial:

*‘I certainly think that general practice could do with a little bit more training to understand, so that they have the ability to train reception staff generally on how to meet the needs of people with ADHD*.*’*

(GP-2)

#### Improving provision

Participants discussed a range of ways that support for ADHD in primary care could be improved. Flexibility was highlighted by many HCPs, including varied ways to book appointments/contact patients:

*‘Flexibility all the way, always to be flexible with the patients. Our senior GP, he is really aware of these patients and is very flexible with them. But he has boundaries with them as well, we’ve still got to have the boundaries*.*’*

(PracticeManager-2)

Meanwhile, many participants with lived experience expressed a need for simpler processes, for example, when booking appointments or ordering medication:

*‘And no more complicated than it has to be. If it can be one document you send in, or like I have my repeat prescriptions, where I don’t even have to type in the medication, I just click “Add”. Just anything to make it easier is wonderful*.*’*

(YoungPerson-2)

Other accommodations mentioned included longer appointments, appointment reminders, performing multiple health-related tasks in one appointment to reduce the need for attendance, and staff continuity:

*‘Don’t let them leave without having a blood test … they might have come in about a form for university, but before they leave we must try and do a medication review, get all of those things done, because we know this might be the only opportunity to see them for a few months*.*’*

(PracticeManager-4)

*‘Even if it’s a couple of GPs that deal with kids with ADHD, well, even adults, so it’s not a different doctor every time*.*’*

(Supporter-2)

## Discussion

### Summary

**Figure 1.**
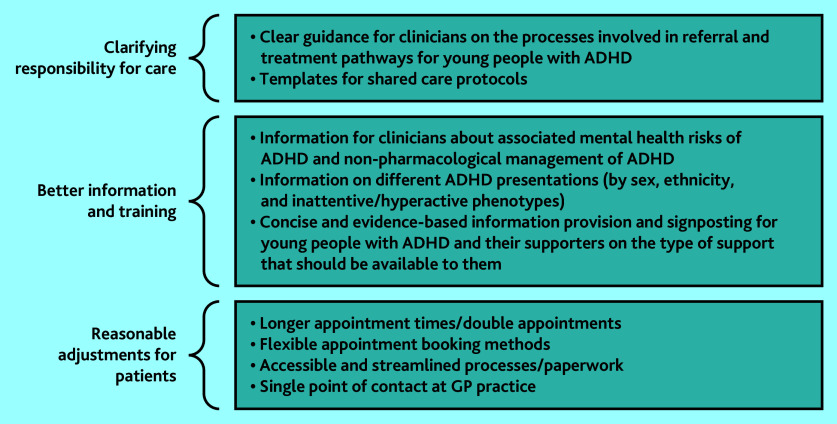
Key recommendations for practice. ADHD = attention deficit hyperactivity disorder.

Our findings evidence the broad range of challenges people living with ADHD and HCPs experience with regards to ADHD management in primary care, including variability in practice and limited resources. These result in individual and systemic stress.^13^ Participants suggested that optimal treatment of ADHD in primary care requires reasonable adjustments for patients and established processes, like those used for other chronic health conditions. The data presented here provide important insights to inform practical and feasible improvements to better integrate healthcare provision and reduce health inequalities for this underserved group.^26^ Figure 1 summarises key recommendations for practice.

### Strengths and limitations

This novel study provides a unique perspective on primary care for young people with ADHD from five diverse sites across England, providing insights into current practice from a range of stakeholders. The sites were chosen to include a range of practice types and populations. For example, the university practice had notable differences in reality compared with practices from areas with high levels of deprivation. The diversity of professional/experiential perspectives captured was also a strength. This research bridges a gap in the literature, providing data on the experiences of people aged 16–25 years, an otherwise under-represented group. An additional strength of this research is that the analysis was conducted with researchers from different professional and experiential backgrounds (psychiatry, general practice, allied health professionals, applied health research, and lived experience), enhancing the credibility and trustworthiness of our findings.^27^

The study has some limitations. Recruiting participants who had previously expressed interest in ADHD research may have resulted in a biased sample, with HCPs with an increased awareness of ADHD and patients with lived experience with polarised experiences of primary care being more likely to take part. Participants varied in sex; however, we did not achieve an ethnically diverse sample of participants with lived experience, thus additional measures to reach underserved groups could be used in the future, such as reaching out to minority participants via community organisations. However, their experiences and reflections remain valid and of interest. Furthermore, our study would have benefited from a broader selection of HCPs (for example, to include social prescribers). This area would benefit from further investigation, especially as roles funded through the Additional Roles Reimbursement Scheme are becoming more widespread in primary care.^28^

### Comparison with existing literature

Our findings concur with extensive literature outlining the pressures faced by services for ADHD across high-income countries,^29^ including the UK,^13^^,^^30^ particularly from a primary care perspective.^31^^–^33 This study demonstrates through stakeholder interviews at five diverse practices across England that the situation is complex, with barriers for both primary care providers and patients. The research has identified challenges including lack of provision; uncertainties in responsibility for care; and variations in provision and care pathways, medication under shared care, and gatekeeper support. Experiences of variation in practices is particularly concerning in light of the fact that ADHD prevalence is higher in deprived areas,^34^ and that under-represented groups with ADHD are least likely to receive appropriate health care for ADHD.^34^^–^36 If GPs are not supported in their role as gatekeepers to secondary care and providing ADHD treatment under shared care, as laid out in NICE guidelines,^11^ then the most vulnerable patients are unlikely to access treatment, increasing health inequalities.

The complexity of systems and processes involved make ADHD management in primary care frustrating and difficult for both patients and clinicians. Our findings resonate with evidence indicating that shared care and communication between primary and secondary services,^15^ concerns for GPs around prescribing a controlled substance,^33^ and complex care pathways^17^ are barriers to care. These barriers would be challenging for any population but are magnified for patients with executive function difficulties, who, for example, often depend on supporters to help them book and attend appointments.^37^ Previous research has outlined the impact that attempting to navigate barriers to health care has on the wellbeing of young people with ADHD.^38^ These individual consequences occur in addition to high costs for society of failing to treat ADHD,^1^^,^^39^ and the additional strain on GPs who already feel burdened by having to find workarounds when appropriate specialist supervision is not in place.^33^

Our findings highlight three areas of focus for improving ADHD provision, all building on evidence from previous qualitative and quantitative research: clarifying responsibility for care,^12^^,^^40^ better information and training,^17^^,^^41^^–^43 and adaptations for people with ADHD in line with the Equality Act 2010.^44^ HCPs and participants with lived experience highlighted that ADHD is often treated differently from other common long-term illnesses, with fewer patient reviews and varying knowledge among practice staff. Thus, there is a need for clear and established protocols in line with those for other conditions, linking with growing recommendations from previous research for mainstreaming of ADHD care^12^ and better organisation of services.^40^ Despite calls for better training for GPs,^16^^,^^17^ this must be considered within the context of the pressures on primary care. Our findings reflect a need for better-quality information in the primary care environment, including about local care pathways and awareness of how symptoms present in under-represented groups. This links with the recognised importance of information in treatment of ADHD, which was added to the NICE guidelines in 2018.^45^ One potentially efficient route towards meeting these needs would be to work with HCPs and people with lived experience to co-design digital health interventions for ADHD, for use in primary care. Our findings emphasise the need to consider non-clinical practice staff, such as receptionists, and their role in increasing accessibility for patients with diverse needs. Finally, accommodations addressing the needs of this patient group, such as longer appointments, frequent reminders, and a flexible approach were mentioned by HCPs and participants with lived experience, in line with NICE guidance, which emphasises the importance of adjustments to support people with ADHD in accessing care.^11^^,^^46^

### Implications for research and practice

Our findings highlight major systemic barriers beyond individual GP awareness and knowledge of ADHD, which affect access to care for young people. Introducing robust and standardised guidelines for the management of ADHD in primary care could contribute to improving access, experience, and outcomes, and to providing clarity for professionals and patients. Additionally, better information and support should be made available to HCPs and other practice staff, including reception and admin teams. Providing information on the diverse ways in which ADHD presents (by sex, ethnicity, and inattentive/hyperactive phenotypes) may help primary care professionals to better support their patients. Finally, reasonable adjustments at practice level, such as offering longer appointments and simplifying administration processes, could help meet the requirements of the Equality Act 2010^44^ by making health care more accessible to individuals living with the attentional and organisational challenges related to ADHD, as well as benefiting patient access more widely.

Future research should focus on evaluating reasonable adjustments that would benefit patients with ADHD, with the aim of producing a template of adjustments, standardised resources, and digital information tools that may support better access to care. Additionally, research that attempts to understand which models of adult ADHD provision in primary care are most beneficial and cost-effective would be of benefit to the evidence base.

## References

[1] Faraone SV, Banaschewski T, Coghill D (2021). The World Federation of ADHD International Consensus Statement: 208 evidence-based conclusions about the disorder. Neurosci Biobehav Rev.

[2] Fayyad J, Sampson NA, Hwang I (2017). The descriptive epidemiology of DSM-IV Adult ADHD in the World Health Organization World Mental Health Surveys. Atten Defic Hyperact Disord.

[3] Song P, Zha M, Yang Q (2021). The prevalence of adult attention-deficit hyperactivity disorder: a global systematic review and meta-analysis. J Glob Health.

[4] Sayal K, Prasad V, Daley D (2018). ADHD in children and young people: prevalence, care pathways, and service provision. Lancet Psychiatry.

[5] Di Lorenzo R, Balducci J, Poppi C (2021). Children and adolescents with ADHD followed up to adulthood: a systematic review of long-term outcomes. Acta Neuropsychiatr.

[6] Shaw M, Hodgkins P, Caci H (2012). A systematic review and analysis of long-term outcomes in attention deficit hyperactivity disorder: effects of treatment and non-treatment. BMC Med.

[7] Huang K-L, Wei H-T, Hsu J-W (2018). Risk of suicide attempts in adolescents and young adults with attention-deficit hyperactivity disorder: a nationwide longitudinal study. Br J Psychiatry.

[8] Chang Z, D’Onofrio BM, Quinn PD (2016). Medication for attention-deficit/hyperactivity disorder and risk for depression: a nationwide longitudinal cohort study. Biol Psychiatry.

[9] Cortese S, Adamo N, Del Giovane C (2018). Comparative efficacy and tolerability of medications for attention-deficit hyperactivity disorder in children, adolescents, and adults: a systematic review and network meta-analysis. Lancet Psychiatry.

[10] Catalá-López F, Hutton B, Núñez-Beltrán A (2017). The pharmacological and non-pharmacological treatment of attention deficit hyperactivity disorder in children and adolescents: a systematic review with network meta-analyses of randomised trials. PLoS One.

[11] National Institute for Health and Care Excellence (2019). Attention deficit hyperactivity disorder: diagnosis and management NG87.

[12] Asherson P, Leaver L, Adamou M (2022). Mainstreaming adult ADHD into primary care in the UK: guidance, practice, and best practice recommendations. BMC Psychiatry.

[13] Young S, Asherson P, Lloyd T (2021). Failure of healthcare provision for attention-deficit/hyperactivity disorder in the United Kingdom: a consensus statement. Front Psychiatry.

[14] Janssens A, Eke H, Price A (2020). The transition from children’s services to adult services for young people with attention deficit hyperactivity disorder: the CATCh-uS mixed-methods study.. Health Serv Deliv Res.

[15] Carrington IM, McAloon J (2018). Why shared-care arrangements for prescribing in attention deficit hyperactivity disorder may not be accepted. Eur J Hosp Pharm.

[16] Tatlow-Golden M, Prihodova L, Gavin B (2016). What do general practitioners know about ADHD? Attitudes and knowledge among first-contact gatekeepers: systematic narrative review. BMC Fam Pract.

[17] French B, Perez Vallejos E, Sayal K, Daley D (2020). Awareness of ADHD in primary care: stakeholder perspectives. BMC Fam Pract.

[18] Price A, Ford T, Janssens A (2020). Regional analysis of UK primary care prescribing and adult service referrals for young people with attention-deficit hyperactivity disorder. BJPsych Open.

[19] Price A, Smith JR, Mughal F (2023). Protocol for the mixed methods, Managing young people (aged 16–25) with Attention deficit hyperactivity disorder in Primary care (MAP) study: mapping current practice and co-producing guidance to improve healthcare in an underserved population. BMJ Open.

[20] Braun V, Clarke V (2006). Using thematic analysis in psychology. Qual Res Psychol.

[21] Byrne D (2022). A worked example of Braun and Clarke’s approach to reflexive thematic analysis. Qual Quant.

[22] McEvoy P, Richards D (2006). A critical realist rationale for using a combination of quantitative and qualitative methods. J Res Nurs.

[23] Office for National Statistics 2021 Census profile for areas in England and Wales. https://www.nomisweb.co.uk/sources/census_2021/report.

[24] Department for Environment, Food and Rural Affairs (2023). 2011 Rural Urban Classification lookup tables for all geographies. https://www.gov.uk/government/statistics/2011-rural-urban-classification-lookup-tables-for-all-geographies.

[25] Ministry of Housing, Communities and Local Government (2019). English indices of deprivation 2019. https://www.gov.uk/government/statistics/english-indices-of-deprivation-2019.

[26] Bisset M, Brown LE, Bhide S (2023). Practitioner Review: It’s time to bridge the gap — understanding the unmet needs of consumers with attention-deficit/hyperactivity disorder — a systematic review and recommendations. J Child Psychol Psychiatry.

[27] Henwood KL, Pidgeon NF (1992). Qualitative research and psychological theorizing. Br J Psychol.

[28] NHS England, NHS Improvement (2019). Network Contract Directed Enhanced Service: Additional Roles Reimbursement Scheme guidance.

[29] Coghill D, Banaschewski T, Cortese S (2023). The management of ADHD in children and adolescents: bringing evidence to the clinic: perspective from the European ADHD Guidelines Group (EAGG). Eur Child Adolesc Psychiatry.

[30] Wright N, Moldavsky M, Schneider J (2015). Practitioner Review: Pathways to care for ADHD — a systematic review of barriers and facilitators. J Child Psychol Psychiatry.

[31] McCarthy S, Wilton L, Murray ML (2012). The epidemiology of pharmacologically treated attention deficit hyperactivity disorder (ADHD) in children, adolescents and adults in UK primary care. BMC Pediatr.

[32] Khan N (2023). ADHD and the rise of the private diagnosis. Br J Gen Pract.

[33] Newlove-Delgado T, Blake S, Ford T, Janssens A (2019). Young people with attention deficit hyperactivity disorder in transition from child to adult services: a qualitative study of the experiences of general practitioners in the UK. BMC Fam Pract.

[34] Prasad V, West J, Kendrick D, Sayal K (2019). Attention-deficit/hyperactivity disorder: variation by socioeconomic deprivation. Arch Dis Child.

[35] Young S, Adamo N, Ásgeirsdóttir BB (2020). Females with ADHD: an expert consensus statement taking a lifespan approach providing guidance for the identification and treatment of attention-deficit/hyperactivity disorder in girls and women. BMC Psychiatry.

[36] Slobodin O, Masalha R (2020). Challenges in ADHD care for ethnic minority children: a review of the current literature. Transcult Psychiatry.

[37] Swift KD, Hall CL, Marimuttu V (2013). Transition to adult mental health services for young people with attention deficit/hyperactivity disorder (ADHD): a qualitative analysis of their experiences. BMC Psychiatry.

[38] Price A, Janssens A, Woodley AL (2019). Review: experiences of healthcare transitions for young people with attention deficit hyperactivity disorder: a systematic review of qualitative research. Child Adolesc Ment Health.

[39] Daley D, Jacobsen RH, Lange A-M (2019). The economic burden of adult attention deficit hyperactivity disorder: a sibling comparison cost analysis. Eur Psychiatry.

[40] Coghill DR (2017). Organisation of services for managing ADHD. Epidemiol Psychiatr Sci.

[41] Price A, Mitchell S, Janssens A (2022). In transition with attention deficit hyperactivity disorder (ADHD): children’s services clinicians’ perspectives on the role of information in healthcare transitions for young people with ADHD. BMC Psychiatry.

[42] Price A, Newlove-Delgado T, Eke H (2019). In transition with ADHD: the role of information, in facilitating or impeding young people’s transition into adult services. BMC Psychiatry.

[43] French B, Sayal K, Daley D (2019). Barriers and facilitators to understanding of ADHD in primary care: a mixed-method systematic review. Eur Child Adolesc Psychiatry.

[44] HM Government Equality Act 2010. https://www.legislation.gov.uk/ukpga/2010/15/contents.

[45] National Institute for Health and Care Excellence (2018). Attention deficit hyperactivity disorder (update): [B] evidence reviews for information and support for people with ADHD. NICE guideline NG87. Evidence review.

[46] Dalrymple RA, Maxwell LM, Russell S, Duthie J (2020). NICE guideline review: attention deficit hyperactivity disorder: diagnosis and management (NG87). Arch Dis Child Educ Pract Ed.

